# Efficiency of Fe_3_O_4_ Nanoparticles with Different Pretreatments for Enhancing Biogas Yield of Macroalgae *Ulva intestinalis* Linnaeus

**DOI:** 10.3390/molecules26165105

**Published:** 2021-08-23

**Authors:** Ahmed El Nemr, Mohamed A. Hassaan, Marwa R. Elkatory, Safaa Ragab, Antonio Pantaleo

**Affiliations:** 1 Marine Pollution Lab, National Institute of Oceanography and Fisheries (NIOF), Alexandria 21556, Egypt; ahmedmoustafaelnemr@yahoo.com (A.E.N.); safaa_ragab65@yahoo.com (S.R.); 2 Advanced Technology and New Materials Research Institute, City for Scientific Research and Technological Applications, Alexandria 21934, Egypt; marwa_elkatory@yahoo.com; 3 Department of Agriculture and Environmental Sciences, Bari University, 70121 Bari, Italy; antonio.pantaleo@uniba.it

**Keywords:** biogas, macroalgae, Fe_3_O_4_, nanoparticles, ozonation, sonication, microwave

## Abstract

In this work, different pretreatment methods for algae proved to be very effective in improving cell wall dissociation for biogas production. In this study, the *Ulva intestinalis* Linnaeus (*U. intestinalis*) has been exposed to individual pretreatments of (ultrasonic, ozone, microwave, and green synthesized Fe_3_O_4_) and in a combination of the first three mentioned pretreatments methods with magnetite (Fe_3_O_4_) NPs, (ultrasonic-Fe_3_O_4_, ozone-Fe_3_O_4_ and microwave-Fe_3_O_4_) in different treatment times. Moreover, the green synthesized Fe_3_O_4_ NPs has been confirmed by FTIR, TEM, XRD, SEM, EDEX, PSA and BET. The maximum biogas production of 179 and 206 mL/g VS have been attained when *U. intestinalis* has been treated with ultrasonic only and when combined microwave with Fe_3_O_4_ respectively, where sediment were used as inoculum in all pretreatments. From the obtained results, green Fe_3_O_4_ NPs enhanced the microwave (MW) treatment to produce a higher biogas yield (206 mL/g VS) when compared with individual MW (84 mL/g VS). The modified Gompertz model (*R*^2^ = 0.996 was appropriate model to match the calculated biogas production and could be used more practically to distinguish the kinetics of the anaerobic digestion (AD) period. The assessment of XRD, SEM and FTIR discovered the influence of different treatment techniques on the cell wall structure of *U. intestinalis*.

## 1. Introduction

Due to their high polysaccharide content and low lignin concentration, macroalgae (seaweeds) have tremendous potential as a feedstock for bioenergy production [1,2]. Large, multicellular sea organisms abound in nature, accounting for over half of the world’s biomass population [3,4]. Seaweeds fix atmospheric CO_2_ for photosynthesis and can multiply quickly, due to a 4-fold higher photosynthetic efficiency than terrestrial biomass [5]. In the year 2000, 11.4 million wet tones of seaweed were collected globally [6]. Pretreatment strategies have been investigated to solve the problem of low CH_4_ productivity. These approaches improve organic matter bioavailability for microbial hydrolysis, reducing hydraulic retention time (HRT) and enhancing biogas production [7,8].

Pretreatment is a common strategy for speeding up the AD process and increasing biomethane production by making previously inaccessible substrates accessible to microorganisms and speeding up the substrate conversion process. The effects of a pretreatment on a particular substrate depend not only on the pretreatment mechanism but also on the characteristics of the substrate [9,10,11].

Chemical pretreatment (oxidative pretreatment) with hydrogen peroxide or ozone (O_3_) has a similar effect on lignocellulose as alkaline pretreatment in that it can also break down lignin. Furthermore, Nguyen et al. [12] reported that O_3_ pre-oxidation of microalgae might cause cell lysis and, as a result, release of intracellular organic materials. Green-blue O_3_ has also been shown to degrade efficiently. Microwave (MW) pretreatment involves employing brief electromagnetic waves with frequencies ranging from 0.3 to 300 GHz to rapidly heat the water in biomass to a boiling state, hence producing pressure within the cells that breaks hydrogen bonds [13,14]. MW pretreatment has a minor influence on biomass solubility [15,16], which holds crystalline cellulose and lignocellulose complexes together, causing the biomass to swell [17]. Sonication waves are supplied to the microalgae culture at ultrasonic frequencies (above 20 kHz). The waves created a succession of micro-bubble cavitation, which transferred kinetic energy to the cell surface and finally broke the cell walls, allowing carbohydrates and lipids to be released into the exocellular media. Ultrasound has been found to degrade microbiological biomass, not lignocellulosic material [18], while some evidence suggests that it improves cellulose accessibility [19]. The breakdown of cells releases hydrolytic enzymes, which aids in increasing the rate of biomass hydrolysis [20,21,22].

Wu et al. [16] study the effects of different pretreatments (mechanical, US and MW) on improving biogas production of Macroalgae *Fucus vesiculosus* and *Fucus serratus*. Pretreatment can significantly affect biogas production because hydrolysis of the algae cell wall structure is a rate-limiting step in the AD process. In this study, four different pretreatments: mechanical, microwave (600 W, 2 min), ultrasonic (110 V, 15 min), and microwave combined with ultrasonic (600 W, 2 min; 110 V, 15 min) were applied to the seaweed and then co-digested with a biogas plant leachate. The results showed that when compared with only mechanical pretreatment, the ultrasonic, ultrasonic combined with microwave, and microwave pretreatments could obtain increased cumulative methane yields of 167, 185, and 156%, respectively. Furthermore, Hassaan et al. [22] study the effect of ozonation on biogas production from *Ulva lactuca*. The ozonation at various dosages was used in contrast to untreated biomass, and the effect on the performance of subsequent mesophilic AD using two separate inoculums (cow manure and activated sludge) was examined. The findings indicated that, in different studies, ozonation pretreatment showed a substantial increase in biogas yield relative to untreated algae. With an ozone dose of 249 mg O_3_ g^−1^ VS algal for *Ulva lactuca*, the highest biogas output (498.75 mL/g VS) was achieved using cow manure inoculum.

Nowadays, nanoparticles (NPs) are increasingly used in health and energy applications. Additives have become a prominent strategy for improving AD performance [21]. Adding various types of NPs to enhance biogas production and promote AD has been investigated in literature [21,22,23]. The most effective quantities of nanoparticles additives were 1 mg/L Co NPs, 2 mg/L Ni NPs, 20 mg/L Fe NPs, and 20 mg/L Fe_3_O_4_ NPs, and they found that Ni NPs produced the highest significant biogas and methane production when compared to Co, Fe, Fe_3_O_4_ NPs, and the control [24]. According to Wang et al. [25], lower Fe^2+^ concentrations (1.3 and 4.6 g/mL) were shown to increase AD, whereas higher Ag^+^ and Mg^2+^ concentrations (3.3 and 9.8 g/mL, respectively) were found to diminish AD. When the AD of sludge induced by nano zerovalent iron (nZVI), Ag NPs, or MgO NPs was compared to the AD induced by the same amounts of Fe^2+^, Ag^+^, and Mg^2+^, and it was discovered that the released Fe^2+^, Ag^+^, and Mg^2+^ were primarily responsible for the enhancement and/or inhibition impacts of nZVI, Ag NPs, and MgO NPs [25]. Only chemical ZnO NPs, according to Mu et al. [26], have a lowering effect on methane generation. Furthermore, reducing the dosage of ZnO NPs (to less than 6 mg/g TS) had little or no impact on methane generation. Hassaan et al. [27] confirmed that NPs could improve the AD process and promote slurry digestion, resulting in increased biogas production, but only at a specific dosage. Lower concentrations of 5 and 10 g/mL of ZnO NPs encourage production biogas from durum wheat, whereas a greater concentration of 20 g/mL of ZnO NPs inhibits it [27].

Because employing many mechanisms and combined techniques that are usually more efficient than methods that use only one tool is also more complex. For example, Rafique et al. [28] studied the effects of thermal, chemical, and thermochemical pretreatment on dewatered pig manure. At 70 °C, high concentrations of lime (5%) exhibited the most significant increase in gas output, far better than lime alone or heat alone. During batch AD experiments, there was a 78% increase in biogas. The importance of this research comes from the necessity to combine the nanoparticles treatment with different thermal, chemical and physical treatments. This works into two distinct pathways: first, working on enhancing the enzymatic activities and the second work on the degradation of the substrate’s cell wall, which increases the ability of biogas production. Moreover, the evaluation of the impact of different treatments and the cell degradation will also be investigated using FTIR, TGA, SEM, and XRD techniques.

In the present study, we have analyzed the impact of two process parameters on biogas production from the macroalgae *U. intestinalis*. First, this work examined how thermal, physical and chemical treatment and nanoparticles affect *U. intestinalis* to break down biomass and their effect on biogas productivity. Second, the combination of *U. intestinalis* pre-treated with iron NPs for biogas production was evaluated to verify whether different processing methods could affect seaweed biogas production. This is the first study that describes the effect of a mixture of three different treatments, either singly or in conjunction with magnetite NPs, when employing sediments as a source of anaerobic bacteria.

## 2. Materials and Methods

### 2.1. Collection of Green Algae U. intestinalis

*U. intestinalis*, a fresh marine green algae, was hand-collected off the Mediterranean shore of Alexandria, Egypt. The biomass was cleaned several times with seawater, tap water, and distilled water before being used. The clean algae was sun-dried for several days before being oven-dried for 24 h at 50 °C. The biomass was then grinding to obtain a fine and homogeneous powder. The dried samples were milled to a size of about 0.5 mm using (Fritsch, Pulverisette 2, Idar Oberstein, Germany) for 5 min, and the milled seaweed samples were placed in plastic bags and kept at room temperature until further use.

### 2.2. Chemical Analysis of Algae Powder

The dry matter has been calculated. By ashing the ground dried samples overnight in a muffle furnace at 550 °C, the ash content was measured. The elemental analyzer was used to calculate C, H, N and S (elemental analysis Vario Micro Cube, Langen selbode, Germany).

### 2.3. Ozonation Pretreatment of U. intestinalis

Using a 0.2 L cylindrical glass containing 150 mL of *U. intestinalis* algal suspension as the working volume, ozonation pretreatments were carried out at a flow rate of 8.3 mg O_3_ min^−1^, O_3_ was guided into the column via a porous glass sprinkler. Using an O_3_ generator, O_3_ was produced (N 1668 a power: 18 W, Vol AC 220 V/50 HZ). All ozonation experiments were performed at pH 8, since when the pH is higher than 7.0, the O_3_ decomposition rate increases dramatically at room temperature (23 ± 2 °C) due to hydroxyl radical formation and three curing times (t) (10, 15, and 30 min) were checked [12,22].

### 2.4. Sonication (US) Pretreatment of U. intestinalis

Using a 0.2 L cylindrical glass containing 150 mL of *U. intestinalis* algal suspension as the working volume, US pretreatments were carried out at pulse 99 and amplitude 99%. Using an US homogenizers, the pulse sonication effect was produced BY Model CY-500—US Homogenizers for three curing times (t) (10, 15, and 30 min). The ultrasonic frequency was 20 kHz. The horn is made of titanium alloy with variable power output rates to vary the effect of the ultrasonic application. The ultrasound probe was made with 1/4 inch titanium alloy (5.6 mm and 60 mm height) [16].

### 2.5. Microwave Pretreatment of U. intestinalis

Using a 0.25 L polytetrafluoroethylene (PTFE) or Teflon lined hydrothermal autoclave reactor containing 150 mL of *U. intestinalis* algal suspension as the working volume. MW pretreatments were carried out at 1100 watts for two curing times (t) (2, and 4 min) by sharp watts MW system [16].

### 2.6. Green Synthesis of Fe_3_O_4_ Nanoparticles

The magnetite (Fe_3_O_4_) NPs were synthesized according to the following method: where 10 g of the dried *U. intestinalis* were refluxed in 100 mL double-distilled water (DDW) for 3 h. Then, the refluxed solution was filtered, and the filtrate was used as the reducing agent. FeCl_3_·6H_2_O and FeSO_4_·7H_2_O in a 2:1 M ratio were added and sonicated for 10 min. After that, the solution was heated to 80 °C for 10 min while magnetically stirring. Then, at 80 °C and steady stirring, 5 mL *U. intestinalis* extract and 20 mL 1 M NH_4_OH were added drop by drop. The obtained colloidal suspensions were then centrifuged, washed repeatedly with ethanol and then dried at 70 °C for 24 h. The final product was calcined at 550 °C to obtain the Fe_3_O_4_ NPs [29].

### 2.7. Fe_3_O_4_ NPs Pretreatment of U. intestinalis

A stock solution of the Fe_3_O_4_ NPs at a concentration of 1 g/L was prepared by dispersing the nanopowder into Milli Q water (conductivity of 18.2 MU/cm at 25 °C). For shock loading, the generated Fe_3_O_4_ NPs solution was diluted to 5, 10, and 20 mg/L in the current study. All of the tests were carried out in duplicate and the T-test in Microsoft Excel was used to calculate the significant difference between the studies.

### 2.8. Inoculum and Substrates Preparation

Microbial seed was obtained from marine sediment collected from El-Mex pump stations (Lat: 31.12486111 and Long: 29.87916667) at Alexandria, Egypt. As proposed by Santegoeds et al. [30] and Do Nascimento et al. [31], 300 g were introduced to 1200 mL sterilized seawater (pH 7) enriched with volatile fatty acids (VFA) mixture and supplemented with nutrients. The inoculum mixture was sealed after removing O_2_ with N_2_ gas.

### 2.9. Biogas Tests

Laboratory tests were conducted on reactors in similar digesters of cylindrical syringes [27,32,33]. The syringes are reversed directly onto the reactor lid [34,35]. A plastic syringe was used to sample the fuel that was equipped with a three-way valve and re-injected into the waste. In all tests, 100 mL glass syringes were applied. As feedstock, 1.5 g of milled *U. intestinalis* (dried weight) was used. In each syringe, 20 g (wet weight) of sediment was applied to the untreated and treated *U. intestinalis*. For 10 min, the working volume was flushed with N_2_. For each anaerobic degradation set-up, three replicates were performed. Until no apparent methane was produced, the inoculum was pre-incubated for three days. At 37 °C with continuous shaking at 150 rpm, the digesters were incubated. Table 1 offers an overview of the substrates used in batch experiments to estimate the *U. intestinalis* biogas yield.

### 2.10. Characterization and Measurement

The following procedures were used to characterize Fe_3_O_4_ NPs, and *U. intestinalis* samples before and after pretreatments with US, O_3_, and MW: Model V-100 VERTEX70, Germany, Fourier transform infrared (FTIR) spectroscopy (platinum ATR) in the wavenumber range (400–4000 cm^−1^), X-ray diffractograms (XRD) were obtained with a Bruker Meas Srv (D2-208219)/D2-2082019 diffractometer operating at 30 kV, 10 mA, and a Cu tube (=1.54) with a 2Theta (2θ) range of 0 to 100°. For both Fe_3_O_4_ NPs and *U. intestinalis*, the surface structure was examined using a JEOL 6360LA scan electron microscopy (SEM). TERIOS Universal V4.5A TA Instruments (New Castle, DE, USA) performed thermogravimetric analysis (TGA) of the impregnated sample for *U. intestinalis* before and after US, O_3_ and MW pretreatments. The prepared green nanostructure Fe_3_O_4_ was characterized individually by Raman (the sample was exposed to this beam for 1 s at 10 mW power with aperture 25 × 1000 mm, three distinct points were measured and displacement occurred between 100 and 1400 cm^−1^), SEM with energy dispersive X-ray spectroscopy (EDX) detector (used to analyze elemental composition of substances), transmission electron microscope (TEM) (JEOL, Model JSM 6360LA, Tokyo, Japan), PSA (The Malvern Mastersizer 3000 is a compact optical instrument that employs laser diffraction to assess particle size distribution), mean pore diameter, and specific surface area were measured on BELSORP (Mini II, BEL Japan Inc., Osaka, Japan) using the BET method (Brunauer–Emmett–Teller) [36].

### 2.11. Kinetics Study and Statistical Analysis

Numerous researchers have used the nonlinear regression models, and the modified Gompertz Equation (1) was applied to determine the cumulative biogas production [37,38]. The model mainly determines the lag phase, biomethane potential, and the max biogas production rate. The biogas production data and the kinetic parameters were defined under widely recognized Equation (1) [37,38,39].

(1) $$M\;=\mathrm{Pb}\times \exp\left\{-\exp\left[\frac{\mathrm{Rm}.e}{\mathrm{Pb}}\;\left(\lambda -t\right)+1\right]\right\}$$

where Pb is the maximum biogas capacity of the substrate (L/g VS added), t is the duration (day), Rm is the maximum bio-gas rate, and e is 2.7183, where M is the biogas yield (L/g VS added) over time t (days). To compare the accuracy of the researched models estimated using SPSS 20, Origin 2020b, and Excel 2010 methodologies, the coefficient of determination (*R*^2^) and root mean square error (RMSE) for both models were obtained. The standard deviation is interpreted as the RMSE, with a lower RMSE implying a better match between predicted and measured values [37,39].

(2) $$\mathrm{RMSE}=\sqrt{\overset{n}{\underset{i=1}{\sum }}\frac{(\mathrm{PVi}-\mathrm{MVi}{)}^{2}}{n}}$$

where PVi is the estimated biogas volume value, MVi is the measured biogas volume value, and n is the number of measurements.

## 3. Results

### 3.1. Characterization of Green Fe_3_O_4_ NPs

#### 3.1.1. Fourier Transform Infrared Spectra (FTIR)

**Figure 1:**
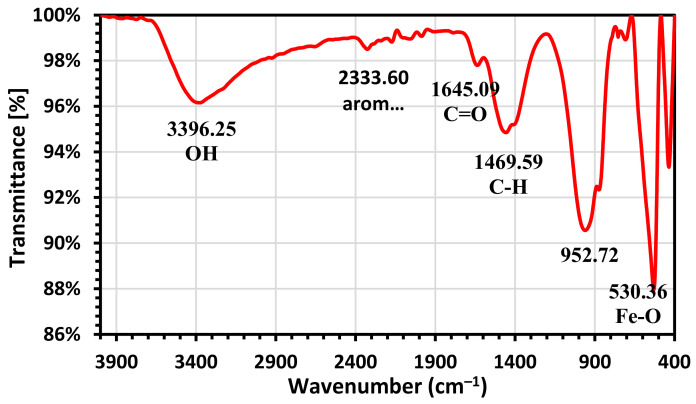
FTIR spectrum of Fe_3_O_4_ NPs.

FT-IR spectroscopy was used to investigate the functionalization of green generated magnetite nanoparticles. In the FT-IR spectra of Fe_3_O_4_ NPs in Figure 1, the absorption peak at 530.36 cm^−1^ is strong. This also shows that the magnetic core is present, as nanoparticles of bare magnetite seem magnetized. The absorption bands 1469.59, 1645.09, and 2333.6 are associated with C–H, C=O, and aromatic components. These bands originate from the extraction of algae. At 3396.25 cm^−1^, the stretching vibration of OH was captured by the band. As a result of hydrolysis on the surface of Fe_3_O_4_, the nanoparticles were hydrated (Fe(OH)_2_, Fe(OH)_3_, and FeOOH) [38,39,40,41]. Therefore, the absorption peak at 436 cm^−1^ indicated that present goethite might be formed by oxidation of Fe(OH)_2_ according to the below reaction in atmosphere in excess NaOH (Equation (3)):

4Fe(OH)_2_ + O_2_ → 4FeOOH + 2H_2_O (3)

#### 3.1.2. Raman Spectroscopy

**Figure 2:**
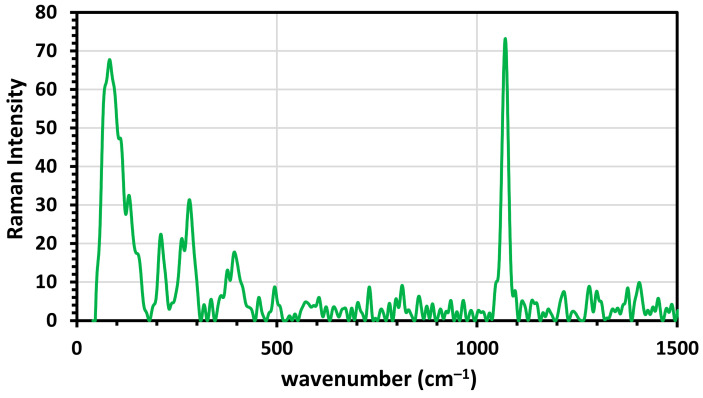
Raman spectrum of Fe_3_O_4_ NPs.

Raman spectroscopy was utilized to identify the iron oxide core (magnetite) type. The Raman spectrum peaks of magnetite were investigated in Figure 2, where five vibrational modes are 212 (T2g(1)), 271 (Eg), 398 (T2g(2)), 493 (T2g(3)) and 659 cm^−1^ (A1g) for the magnetite [42,43,44]. Raman spectrum includes a strong peak located at 385 cm^−1^. Other less intense peaks at 584 and 685 cm^−1^ indicated goethite (FeOOH), and the peak vibrated at 1069 cm^−1^ was related to the organic compound of the capping agent. According to Testa-Anta [45], most metal-oxygen lattice vibrations occur below 750 cm^−1^, the main vibrations of organic molecules occur above 1000 cm^−1^.

#### 3.1.3. X-ray Diffraction (XRD)

**Figure 3:**
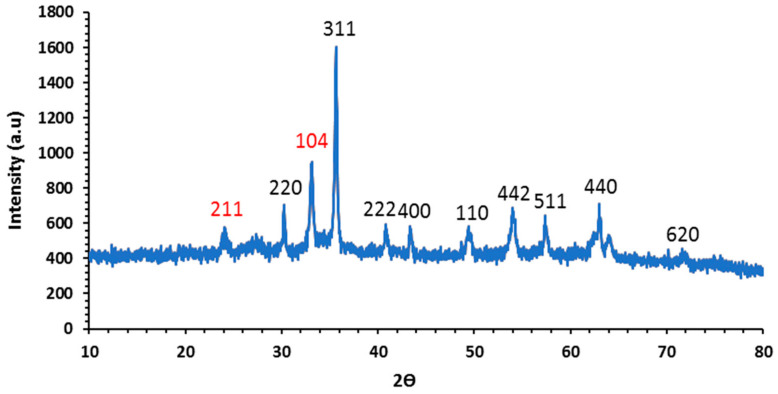
X-ray diffractograms of Fe_3_O_4_ NPs.

X-ray diffraction highlights the formation of magnetite as the major crystalline phase in the sample synthesized by co-precipitation process. The main characteristic peaks of magnetite (Fe_3_O_4_) were identified at 2θ (°) = 30.24, 35.67, 37.46, 40.85, 43.47, 49.39, 54.09, 62.98, and 69.81, which corresponds to the (220), (311), (222), (400), (110), (422), (511), (440) and (620) diffraction plane may be well indexed to the inverse cubic spinel structure of Fe_3_O_4_, (Figure 3). This demonstrates that the Fe_3_O_4_ nanoparticles are generated in this work. The small peak visible at 2θ = 24.17° is attributed to the goethite structure (FeOOH) corresponding to the (110) plane. It is not easy to differentiate these structures even if both phases exhibit high crystallinity. However, some authors report that in the XRD pattern associated with the goethite phase, there exist two additional peaks located at 24.17° and 33.12° (211) and (104) [35].

#### 3.1.4. Scanning Electron Microscopy (SEM)

**Figure 4:**
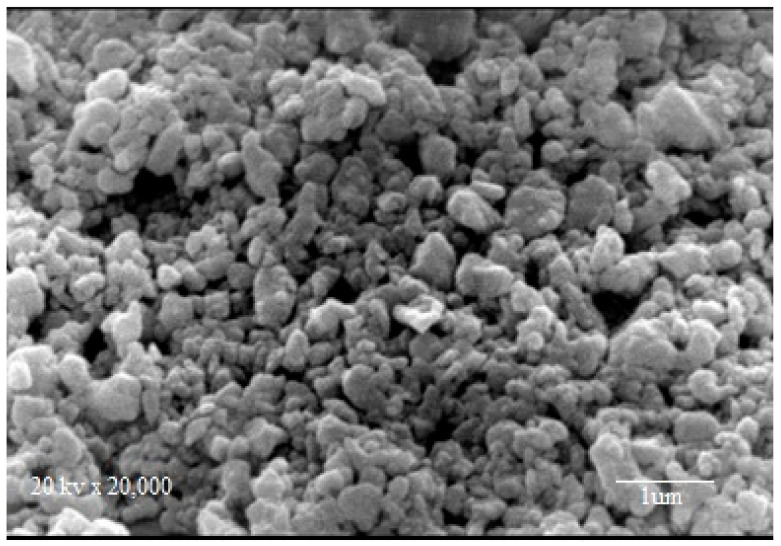
SEM of Fe_3_O_4_ NPs.

The creation of large agglomerates of nanoscale particles can be attributed to the expansion through coalescence of nuclei, resulting in particles that tend to cluster toward a lower energy state free, by reducing interfaces with the environment, as shown in the SEM image (Figure 4). The elemental composition of Fe_3_O_4_ is determined by EDX. Table 2 showed that the iron and oxygen contents are 81.13 and 18.87 mass%, respectively. It can be seen that the oxygen content increases with the presence of goethite (FeOOH) indicating the presence of magnetite as the host material with a small portion of goethite.

#### 3.1.5. Transmission Electron Microscopy (TEM)

**Figure 5:**
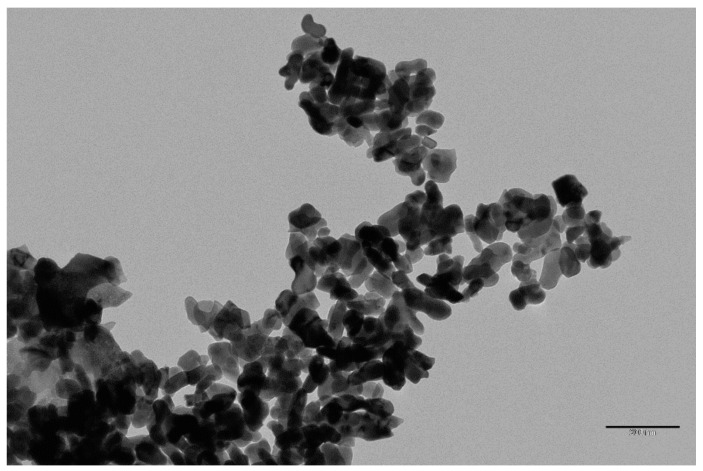
TEM of Fe_3_O_4_ NPs.

TEM micrograph (Figure 5) showed spherical agglomerated particles; agglomeration might be due to solvation and capping of nanoparticles by algal extract [33]. The particle sizes are in the range 5.6–16.8 nm.

#### 3.1.6. Particle Size Analyzer (PSA) and BET Analysis of the Surface Area

**Figure 6:**
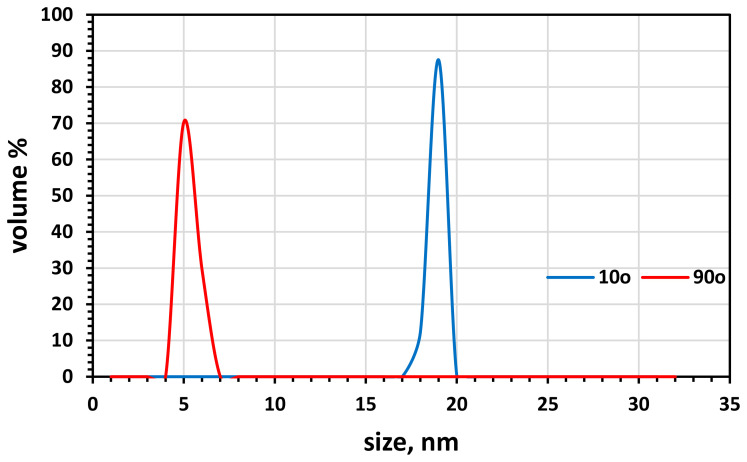
PSA of the magnetite Fe_3_O_4_ NPs.

Figure 6 depicts the PSA-defined particle size distribution for Fe_3_O_4_ NPs. The Fe_3_O_4_ nanoparticles have a uniform particle size, as evidenced by the detection of Fe_3_O_4_ particles in the range of 6 to 8 nm using a 10° test angle and another range of particle size from roughly 200 to 257 nm using a 90° test angle. The green Fe_3_O_4_ NPs’ uniformity and homogeneity may have a good impact on biogas generation. The BET analysis of green Fe_3_O_4_ shows that the synthesized magnetite nanoparticles’ surface area and average pore size were 37.85 m^2^/g and 9.56 nm, Table 3.

### 3.2. Characterization of the Pretreatments Analysis of U. intestinalis

#### 3.2.1. Fourier Transform Infrared Spectra (FTIR)

**Figure 7:**
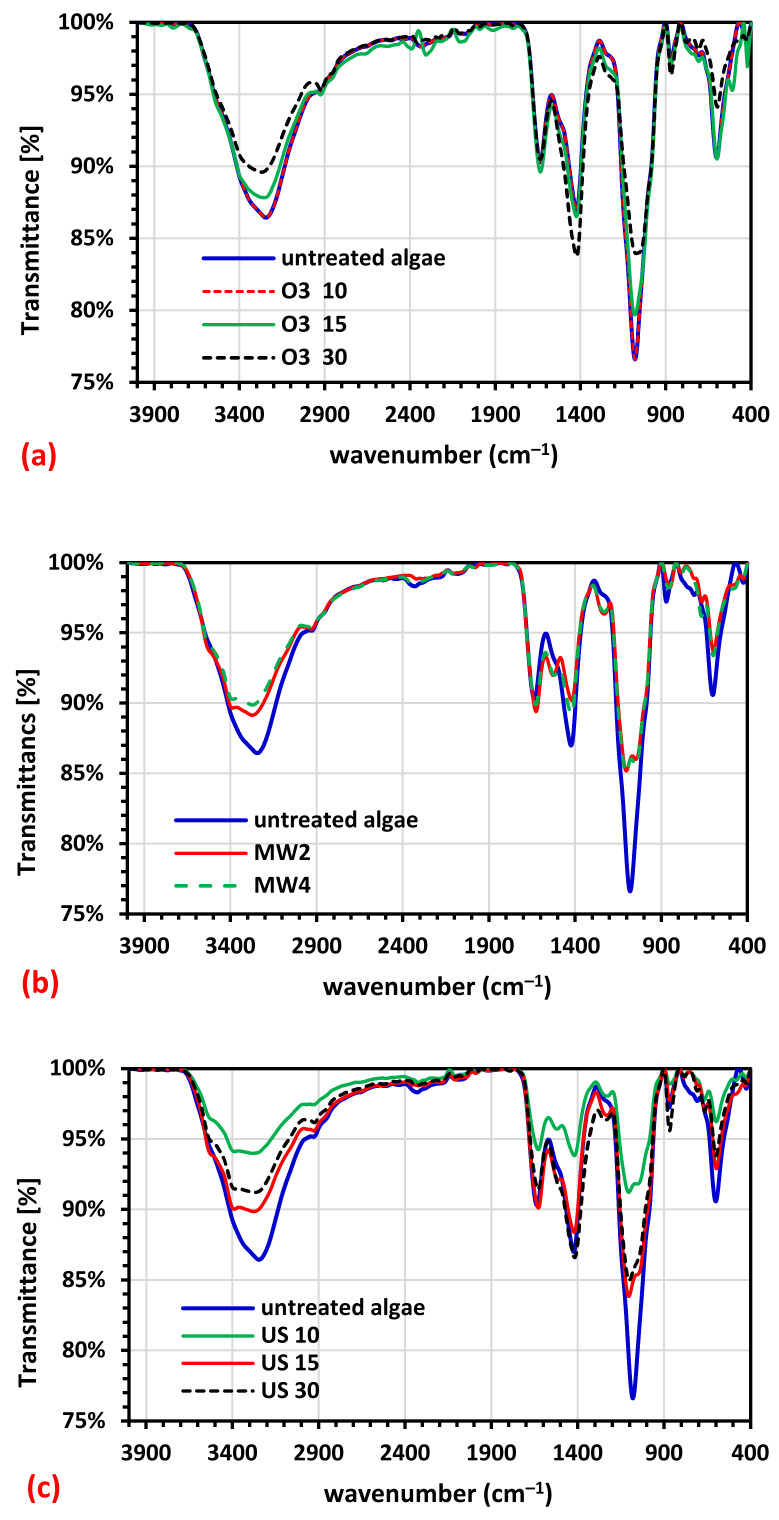
FTIR spectrum of (**a**) raw and ozonated pretreated *U. intestinalis*, (**b**) raw and MW pretreated *U. intestinalis* and (**c**) raw and sonicated pretreated *U. intestinalis*.

Figure 7 depicts the FTIR spectra of *U. intestinalis* biomass before and after pretreatment with US, O_3_, and MW, which show relative peaks at different wavenumbers. This is because, while only water is utilized during pretreatment, the above-mentioned pretreatments do not add new chemical groups to the solid fraction of seaweed following pretreatment. It reveals that there are two absorption peaks in the hydrogen bonding area ranging from 2927 to 3729 cm^−1^. The stretching vibrations of hydrogen-bond O–H and N–H groups are assigned to the primary peak at 3267 cm^−1^, showing phenolic and alcoholic chemicals, carbohydrates, and proteins in the *U. intestinalis* biomass. The vibration of the C–H group in the polysaccharides in the *U. intestinalis* biomass is responsible for the soft peak at 2964 cm^−1^. The C=O group of amides, which arises due to proteins, is ascribed to the peak at 1641 cm^−1^. The peak around 1429 cm^−1^ could be attributed to the aromatic structure’s C–C stretching vibration. The peak at 1083 cm^−1^ is typical of aliphatic amines’ C–N and C–O–C stretching vibrations, indicating the presence of proteins and polysaccharides [46,47]. The FTIR spectra show that raw seaweed displayed high stretching vibration peaks corresponding to the O–H and N–H groups (3454 cm^−1^). However, these absorption strengths decreased in the processed seaweed, suggesting the decomposition of carbohydrates and proteins. The vibration peaks corresponding to the C–H and C–C groups (2964 and 1429 cm^−1^) similarly dropped in a similar manner, which was related to hydrolysis of polysaccharide components after pretreatment with US, O_3_, and MW. The decrease in C=O and C–N vibration peaks (1641 and 1083 cm^−1^) demonstrated that the proteins in the treated seaweed had degraded.

#### 3.2.2. X-ray Diffraction (XRD)

**Figure 8:**
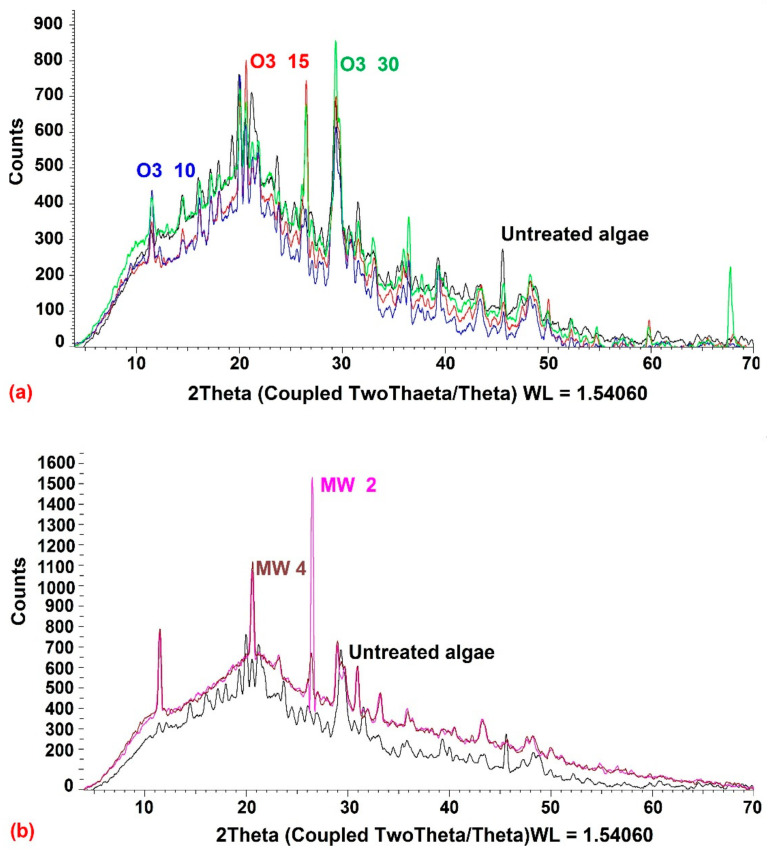

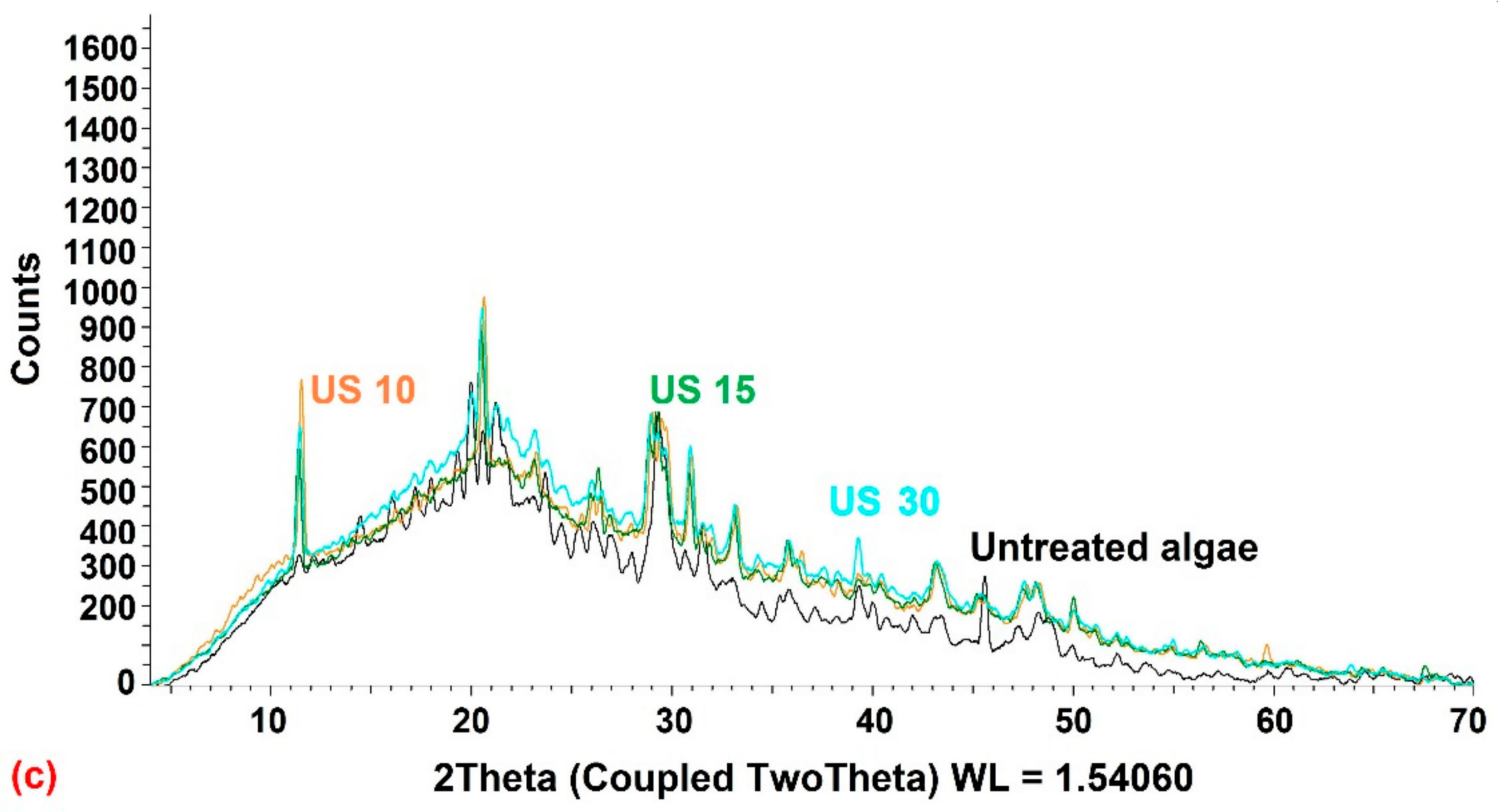
X-ray diffractograms of (**a**) raw and ozonated pretreated *U. intestinalis*, (**b**) raw and MW pretreated *U. intestinalis* and (**c**) raw and sonicated pretreated *U. intestinalis*.

The initial degree of crystallinity is a crucial element to assess the pretreatment process. The crystallinity of raw and pretreated *U. intestinalis* was studied using X-ray diffraction analysis (Figure 8). The crystallography showed that after US, O_3_ and MW pretreatments, the peak strength of the raw *U. intestinalis* sample became sharper at 2, 20, 26, 27, and 30, Figure 8. These peaks tend to conform to crystalline cellulose after pretreatment. This may prove that the pretreated *U. intestinalis* crystallinity increased when pretreated with US, O_3_ and MW.

#### 3.2.3. Thermal Analysis (TGA)

**Figure 9:**
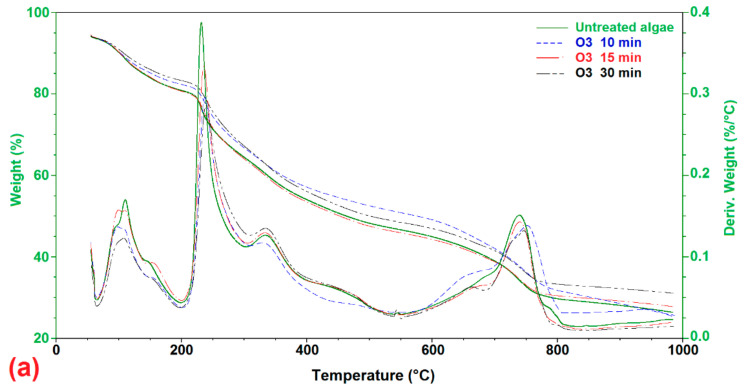

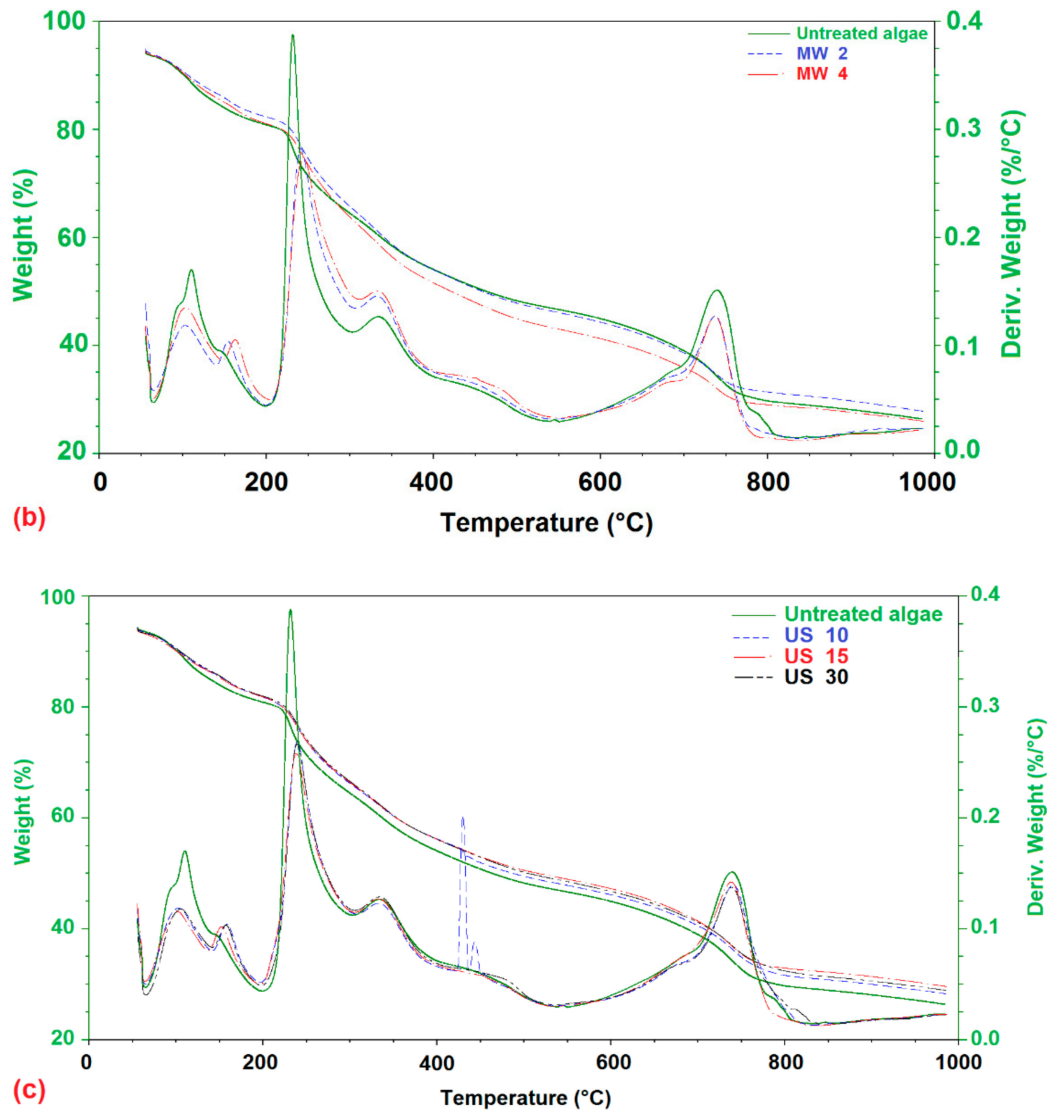
TGA and DTA thermographs of (**a**) raw and ozonated pretreated *U. intestinalis*, (**b**) raw and MW pretreated *U. intestinalis* and (**c**) raw and sonicated pretreated *U. intestinalis*.

According to the findings in Figure 9, the thermal degradation of algae samples occurred in three stages. In the first level, from 70 to 100 degrees Celsius, there was a weight loss, which can be explained by evaporation of the sample’s moisture content [48] or some light volatile materials [49,50,51,52]. On the other hand, the second level took place from 100 °C to a temperature of up to 400 °C. As a result of the significant deterioration process, consequential weight loss was noted at this point. Organic components of algae, such as carbohydrates, protein, and lipids, decompose and/or depolymerize, resulting in this loss. The mass loss of algae between 180–270 °C is due to carbohydrate decomposition, while protein degradation occurs between 320–450 °C, and the third stage linked to lignin decomposition occurs and restart from 500 °C [39]. In our study, the peak in range 626.85–826.85 °C may be due to the macroalgae’s ability to remediate heavy metals by uptaking these metals inside their cells and remains in the *U. intestinalis* after pretreatments because they create cavities and blockage in the outer cell wall [48].

#### 3.2.4. Surface Morphology, Scanning Electron Microscopy (SEM)

**Figure 10:**
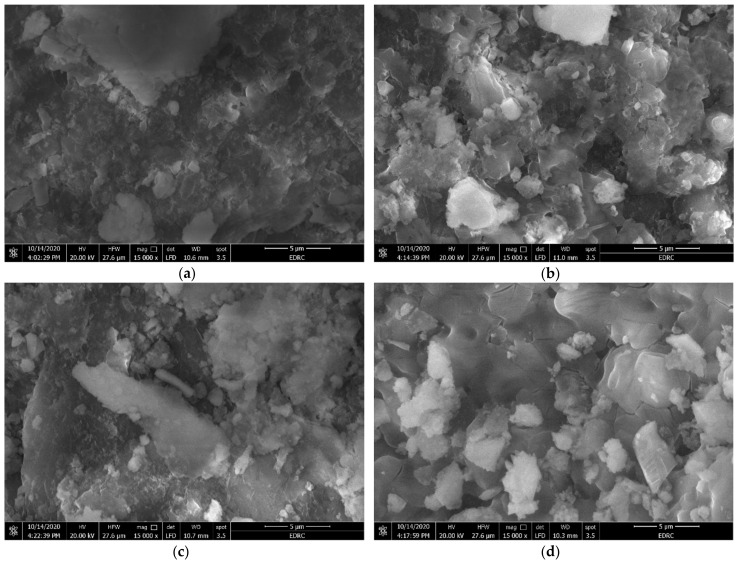
SEM images of (**a**) raw, (**b**) ozonated pretreated *U. intestinalis,* (**c**) MW pretreated *U. intestinalis,* and (**d**) sonicated pretreated *U. intestinalis*.

SEM evaluated the influence of the pretreatments on the structure of macroalgae. The morphology of macroalgae provided an alternative view, giving a better understanding of the effect of using US, O_3_ and MW rather than untreated *U. intestinalis*, to better understand the effect of the pretreatments on the AD of the green macroalgae *U. intestinalis*. Un-treated *U. intestinalis* (Figure 10) indicates a braided canvas with only a few broken fibers. At the same time, the pre-treated samples of *U. intestinalis* showed that it is possible to distinguish broken cell walls and disrupting their integrity. This suggests, therefore, that the pretreatments could harm the algae cell wall and create hydrodynamic cavities with a more superficial degradation and blockages, but the *U. intestinalis* treated with the US was better than O_3_ and MW [23].

### 3.3. Chemical Compositions of U. intestinalis

As shown in Table 4, the VS content of the investigated *U. intestinalis* is 70.55%. On the other hand, by means of an elemental analyzer, the determination of the C and N material is detected and the measurement procedure is followed [50]. Table 4 shows a C/N ratio of about 9.60%. It is also worthy to mention that, the reported literature [53,54,55] indicated that the optimum C/N ratio is 16–19% for better methanogenic efficiency when considering hardly degradable complexes such as lignin [51]. However, this ratio is near the same ration for the present studied biomass *U. intestinalis*.

### 3.4. The Impact of Different Pretreatment Techniques on Anaerobic Digestion and Biogas Production

**Figure 11:**
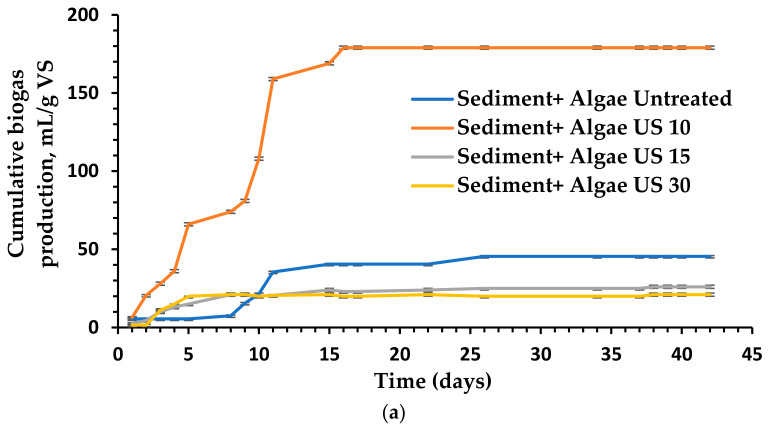

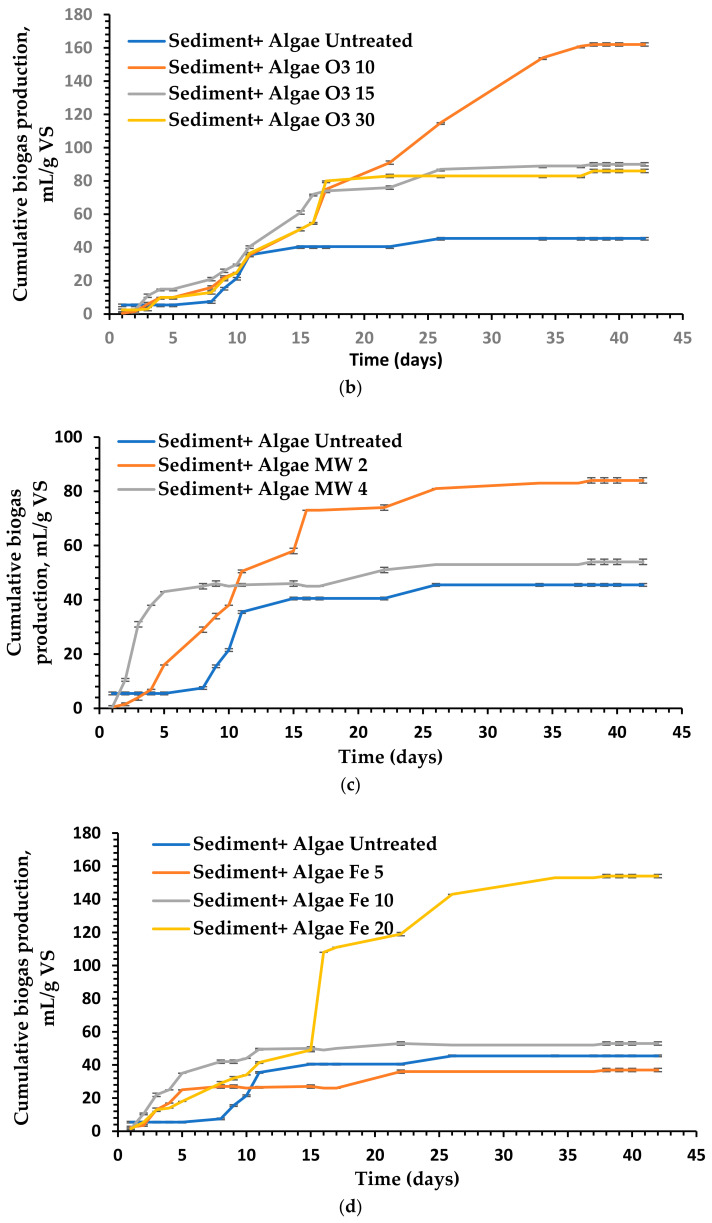

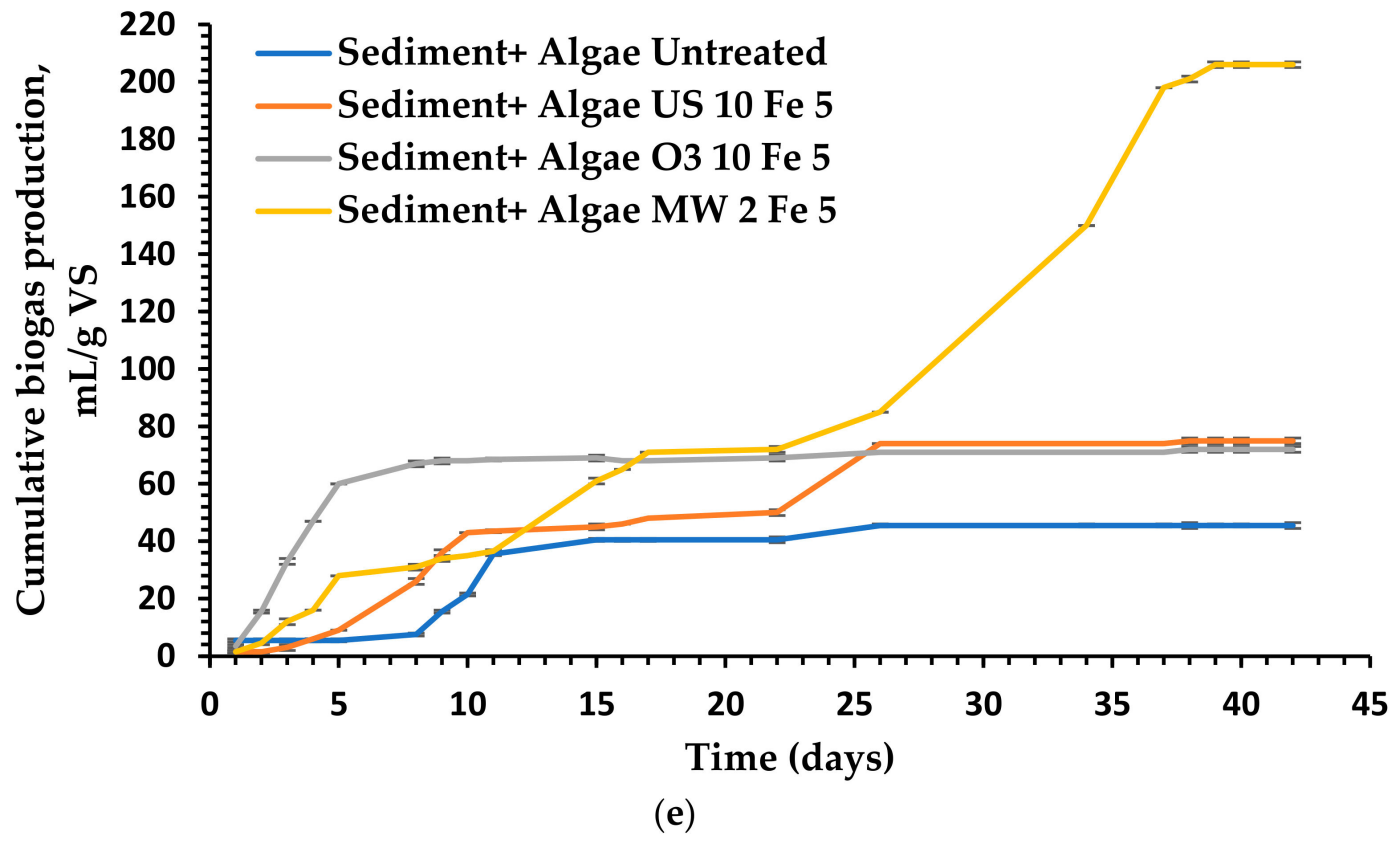
Average production of cumulative net biogas (mL/g VS) using (**a**) raw and sonicated pretreated *U. intestinalis*, (**b**) raw and ozonated pretreated *U. intestinalis*, (**c**) raw and MW pretreated *U. intestinalis*, (**d**) raw and Fe_3_O_4_ NPs *U. intestinalis* and (**e**) raw and combination of different treatment with Fe_3_O_4_ NPs.

**Figure 12:**
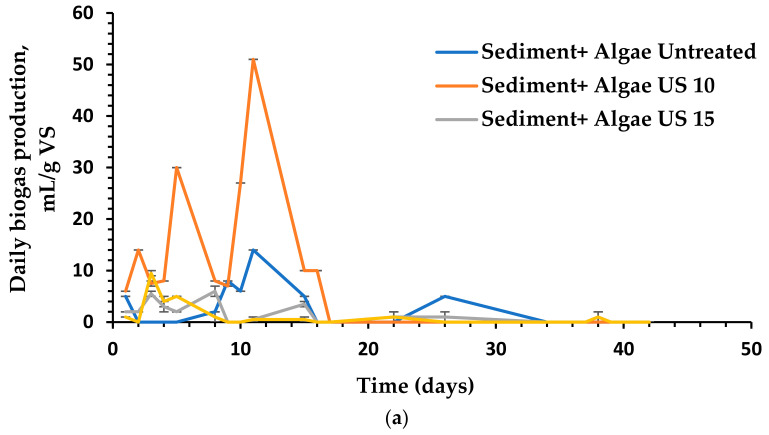

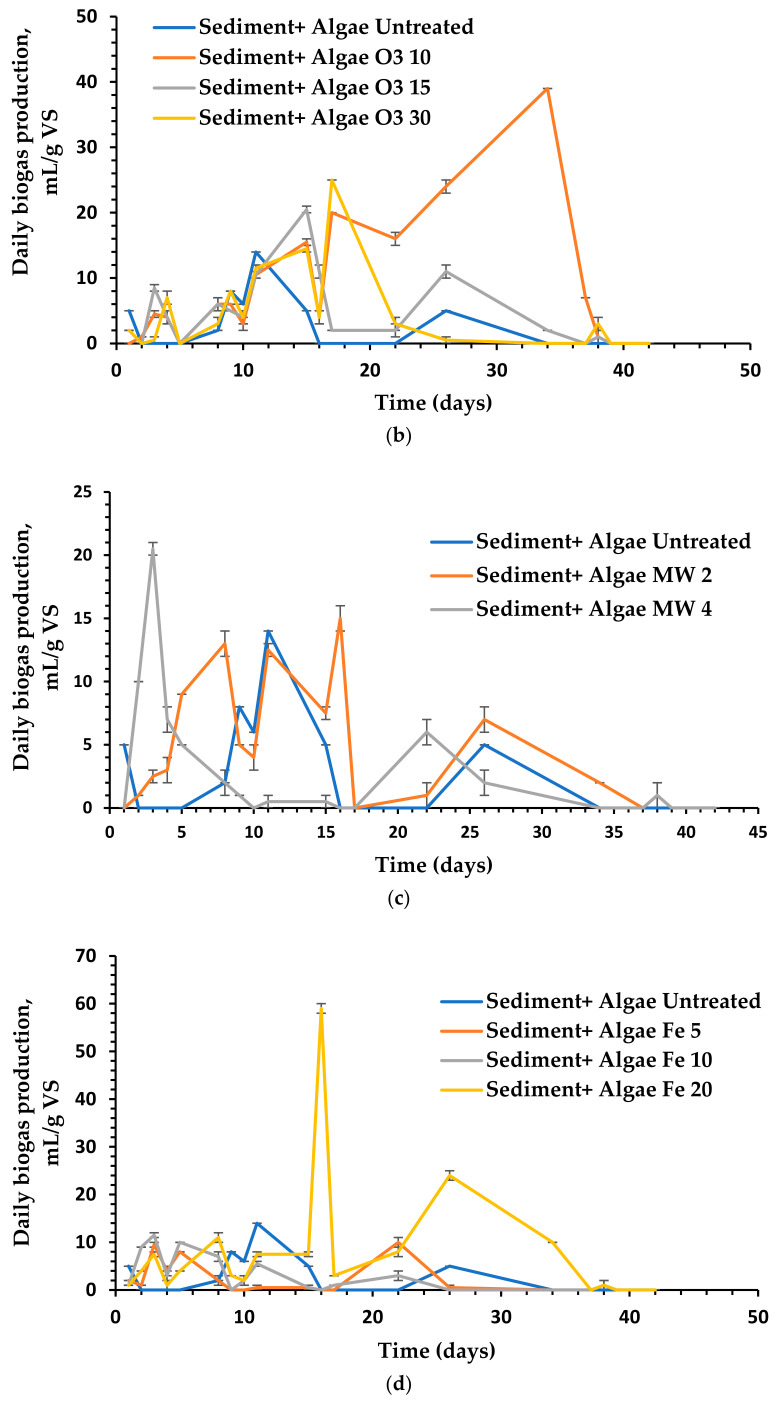

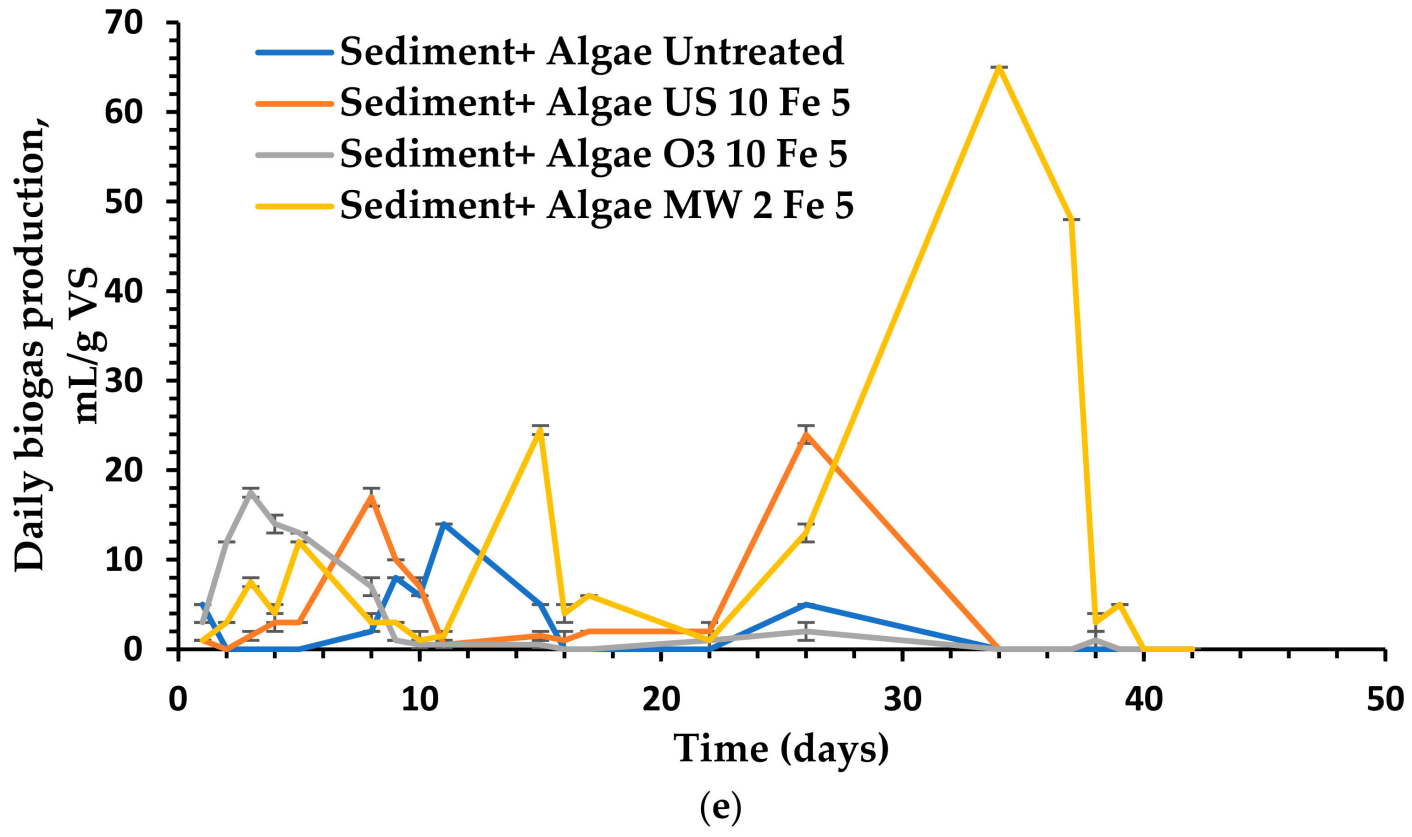
Average daily production of biogas using (**a**) raw and sonicated pretreated *U. intestinalis*, (**b**) raw and ozonated pretreated *U. intestinalis*, (**c**) raw and MW pretreated *U. intestinalis*, (**d**) raw and Fe_3_O_4_ NPs *U. intestinalis* and (**e**) raw and combination of different treatment with Fe_3_O_4_ NPs.

The experimental findings of biogas outputs were collected over a 42-day period, as shown in Figure 11. In the beginning, hydrolysis and fermentation were the main processes, and the biogas yield was relatively low. Following the initial anaerobic process that provided substantial biogas outputs in the first step, inactivity presumably due to the methanogens undergoing a metamorphic growth phase [56,57]. When the *U. intestinalis* was treated with US (pulse 99 and amplitude 99%), O_3_ dose (8.3 mg O_3_ min^−1^ VS) with time intervals of 10, 15 and 30 min and MW (1100 Wt) with time intervals of 2–4 min, the average biogas production yield was marginally increased compared to the biogas production yield obtained from the untreated *U. intestinalis*. The best biogas results production (206 mL/g VS) in this study was attained when the substrate was treated with MW in combination with Fe_3_O_4_ NP with a concentration of 5 mg/L, Figure 11e. This biogas yield is more than two and a half times greater than of the individual MW treatment (84 mL/g VS). Figure 11a, displays the tested US treatment properties as duration (10, 15, and 30 min), demonstrating that US treatment can improve algal cell wall solubility, allowing for enhanced biogas production via anaerobic digestion or acceleration of the anaerobic process. The second better biogas production yield were from those with the shortest US time treatments. In particular, the highest biogas generation (179 mL/g VS) was observed for a US period of 10 min Figure 11a. These values were accepted as the optimal dose in US treatment before anaerobic digestion testing, where they are higher than those generated from untreated mixed anaerobic digestion. Moreover, the higher US time for 15 and 30 min has inhibitory effects on the biogas generation due to the development of less biodegradable by-products than the untreated substrates. Figure 11b,d demonstrates the effect of ozonation and different Fe_3_O_4_ NPs concentration on the biogas production from *U. intestinalis*. The biogas yield was higher than individual MW treatment with biogas yield of (162 and 154 mL/g VS) for O_3_ 10 min and FE_3_O_4_ 5 mg/L treatments, respectively. As seen in Figure 12, the biogas output tests were completed when the regular production of biogas was <1% of the total production of most of the tests conducted.

## 4. Discussion

The usage of US in liquid solutions is based on monolithic cavitation, which has physical and chemical effects [58]. The physical impacts are caused by the collapse of cavitation bubbles, which results in an increased chemical modification due to the creation of free radicals [59]. Microbiological cells can be destroyed, and harmful chemical substances can be oxidized, as a result of these impacts [60,61]. Several studies [62,63,64] allude to the use of sonolysis to increase COD solubility and anaerobic biodegradability of sewage sludge prior to anaerobic digestion in the United States; they demonstrate that sonolysis can greatly improve COD solubility and anaerobic biodegradability of sewage sludge. Kim et al. [65,66] discovered that when US pretreated sludge was compared to untreated sludge, methane generation rose by 34%. Low-frequency degradation of surplus sludge has been demonstrated to be more efficient: mechanical effects increase particle solubility, ensuring the availability of higher amounts of readily digested organic materials in the liquid phase [66,67]. Despite the fact that US generation consumes energy, it has been observed that the US process modifies the organic matter structure, making it significantly more homogeneous and less sedimentary [58]: as a result, less energy (500 W—50 Hz) is required to improve particle solubility in the digesters. However, to determine the practical costs and benefits, additional, comprehensive research in a broader size is needed. In our work, the *U. intestinalis* treated with 10 min ultrasonic produce highest cumulative biogas production 179 mL/g VS, which means that ultrasonic pretreatment could promote the hydrolysis of carbohydrate polymers to reducing sugar.

Lower O_3_ doses have significant positive effect on the production of biogas (*p* < 0.05). The ozonation time of 10 min produces a higher biogas yield with 162 mL/g VS for *U. intestinalis* combined with sediments. Because of the generation of less biodegradable by-products than untreated substrates, biogas quantities produced by O_3_ pretreatment were found to be larger at 10 min than those generated by untreated mixed anaerobic digestion and for treated with O_3_ durations of 15 and 30 min. According to these findings, compared to time 10 min, exposure to O_3_ for longer than 10 min does not determine additional oxidation effects. From the previous results of different biotechnology fields, it is safe to assume that the promise of ozone can also be used in the anaerobic digestion process to enhance the ferment ability of the macroalgae biomass. Hassaan et al. [22] stated that the higher doses of O_3_ (15 and 30 min) increased the biogas ability of the studied green algae *Ulva lactuca*, compared to untreated biomass studies when he uses manure and sludge as a source of bacteria. This variability could be attributed to the source of manure, wherein in our research, we have used sediment, which contains different media for AD. The evaluation of FTIR, TGA, SEM, and XRD in this study are in agreement with the results obtained by Hassaan et al. [22], which revealed the impact of O_3_ on the structure of the algal cell wall and integrity breakage (Figure 10), which was thus established as the main contributor to improving the biogas production.

It is clear that the MW treatment only gives the lowest biogas yield among the studied treatment techniques with 84 mL/g VS after 2 min of treatment. Wu [16] stated that the Microwave pretreatment with a power of 700 W could improve biogas production in the range of 7.8–43.7% when applied for 1.5 min and in the range of 37.2–45.2% when applied for 3 min. However, several studies showed that microwave pretreatment had no or adverse impact on biogas production. The small biogas production through microwave pretreatment may be explained by the change in osmotic pressure and the output from solubilization of lignin through microwave pretreatment, which has detrimental effect on anaerobic bacteria [16]. On the other hand, when comparing the various treatments and the control, the results showed that MW pre-treatment in combination with Fe_3_O_4_ NPs produced the best biogas yields. The amount of biogas produced by the MW pretreatment + Fe_3_O_4_ NPs group was 206 mL/g against the control group. Multiple pretreatment combinations have been examined to improve biomass enzymatic hydrolysis and the corresponding BMP. They cannot be classified as mechanical, thermal, or chemical pretreatment because they involve a combination of methods. While combination pretreatments are more complicated than typical treatment techniques, they are more successful.

Finally, it is worse to mention that when *U. intestinalis* was treated with Fe_3_O_4_ of 5 mg/L it gives 154 mL/g Vs biogas yield which is higher than MW treatment alone and the order of biogas production according to the used techniques can be arranged as the following: MW + Fe_3_O_4_ > US > O_3_ > Fe_3_O_4_ > MW. It is also worth to mention that the little dosage of the treatment give higher biogas yield when we use sediment as manure and this behavior needs more investigation. Low dosage have a significant positive effect on the production of biogas (*p* < 0.05). When compared to untreated *U. intestinalis*, the improvement in combined Fe_3_O_4_ with MW treated *U. intestinalis* and individually sonicated *U. intestinalis* is greater than other NP treatments, such as nano zero valiant iron (nZVI) and Fe_2_O_3_ NPs with concentrations of 10 and 100 mg/g TSS, which produces more cumulative methane at 120 and 117 percent of total biogas yield, respectively [68].

However, addition of the magnetic Fe_3_O_4_ NPs was found to improve biogas production more than untreated algae at 20 mg/L maximum concentration, which is consistent with Abdelsalam et al. [24], who concluded that magnetic NPs appeared to be non-toxic during long-term contact and only exhibited mild toxicity to bacteria at the initial stage. In contrast, our research found that adding 20 mg/L Fe_3_O_4_ magnetic NPs to biogas production during starting and over the first 42 days of HRT increased bacterial activity. According to our findings, the best biogas productivity was produced utilizing 5 mg/L Fe_3_O_4_ magnetic NPs in combination with MW treated macroalgae. These findings showed that Fe_3_O_4_ magnetic nanoparticles improved anaerobic digestion, increasing biogas production and organic matter decomposition. The presence of Fe^2+^/Fe^3+^ ions, which were injected into the reactor as nanoparticles and could be adsorbed as a growth ingredient for anaerobic microbes, increased performance [24].

The physiochemical properties confirm the presence of magnetite (Fe_3_O_4_) and a small amount of goethite (FeOOH), where magnetite release of bioavailable ions (Fe^2+^ and Fe^3+^), which is known as an essential nutrient for microbial power generation. DNA replication [69] and key enzymes formation furthermore, increased concentration of acetate and butyrate which are known as energy favorable volatile fatty acids (VFAs) for the methane production phase [70], may consequently promote the AD process, as well as better effluent quality [71]. Therefore increase microbial abundance and activities of key enzymes or coenzymes. Furthermore, increased concentration of acetate and butyrate, known as energy favorable volatile fatty acids (VFAs) for the methane production phase [70], may consequently promote the AD process and better effluent quality. Serve as conduits for electrons, hence stimulate electron transfer between the bacterial and archaeal communities to reduce carbon dioxide (CO_2_) to CH_4_ [72]. While, the goethite (FeOOH) maybe act as capable of absorbing inhibitory compounds pollutant species, including a high abundance of ammonia, phosphorus and sulphate, together with excessive amounts of heavy metals in wastewater treatment rapidly and thoroughly via precipitation [73,74] and trap these compounds on their surface [75,76]. Generally, both iron oxides (magnetite and goethite) works as a pH buffer, thus stabilize the AD system [75,76]. Finally, all the publications concluded that the influences on the AD process nano-iron-additives were dosage-dependent. An excessive dosage of iron-based nanoparticles hindered the overall process resulting in reductions in biogas production. The results indicated that iron oxide NPs additives have a positive impact and improved biogas production by releasing two electrons due to oxidation to Fe^2+^ under anaerobic conditions [77]. The electrons released by Fe can be consumed by inorganic CO_2_ or acids and accelerated by the hydrogenation pathway and thus produce more CH_4_ [77]. Where the highest specific biogas production (154 mL/g VS) for untreated algae with Fe_3_O_4_ NPs of 20 mg/L.

## 5. Kinetic Study

**Figure 13:**
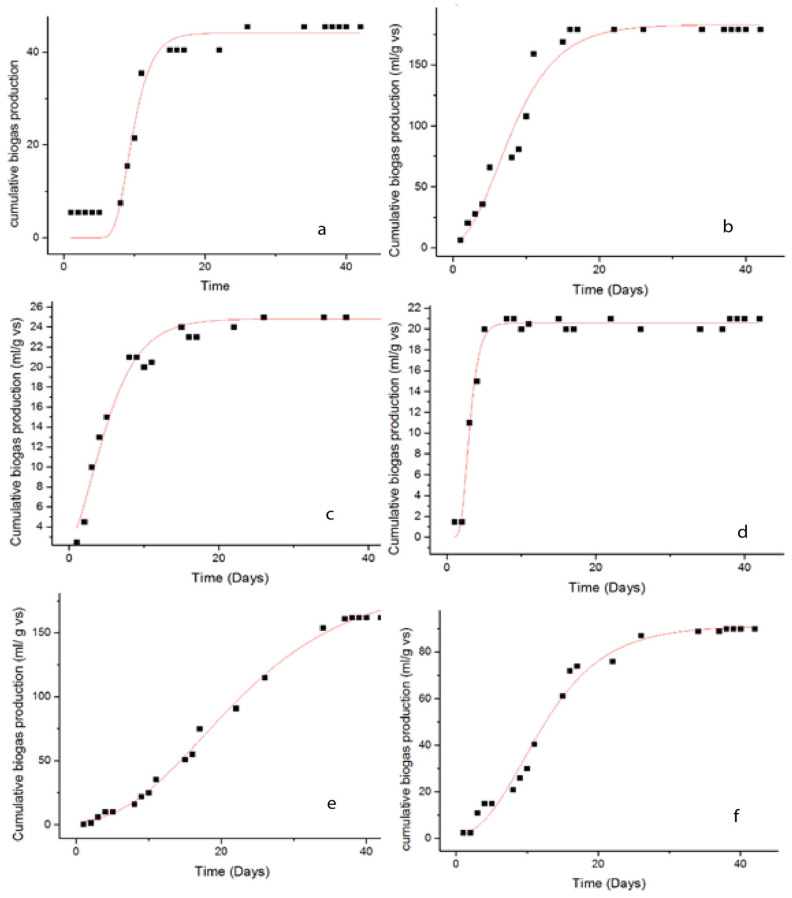

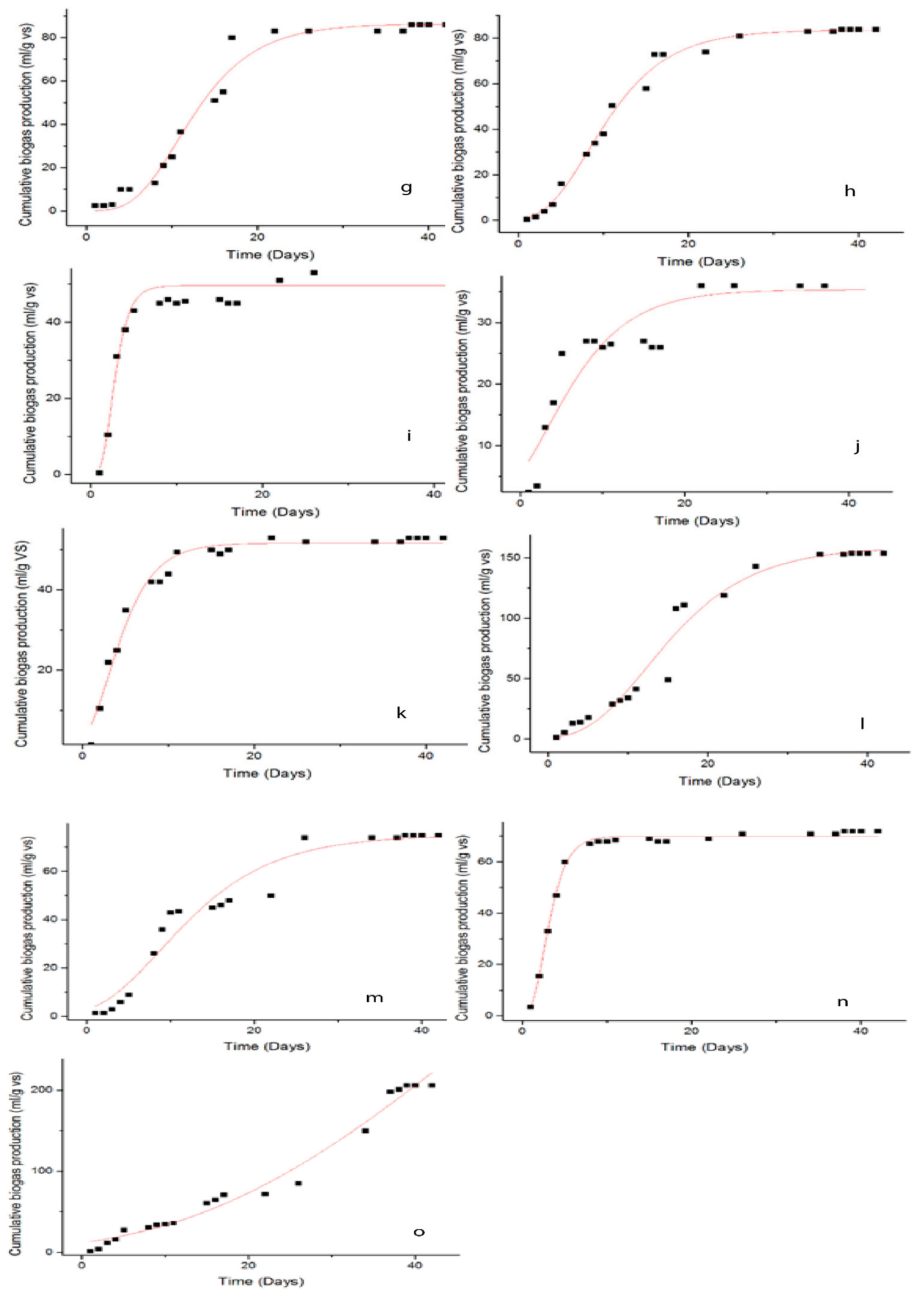
Cumulative biogas yield from Gompertz model, (**a**) untreated, US 10, 15, 30 min (**b**–**d**), O_3_ 10, 15, 30 min (**e**–**g**), MW 2, 4 min (**h**,**i**), Fe_3_O_4_ 5, 10, 20 mg/L (**j**–**l**) and combined US + 5 mg/L, O_3_ + 5 mg/L, MW + 5 mg/L (**m**–**o**).

The modified Gompertz equation has been shown in previous studies to be a standard model for biogas production from a simple organic substrate. The modified Gompertz model was used to fit the cumulative methane yields obtained from the anaerobic co-digestion trials. Figure 13 shows a comparison of fitting results produced from the modified Gompertz model and experimental data. As observed in Table 5, the *R*^2^ values were all greater than 0.9, indicating that the modified Gompertz model performed well in representing the cumulative process of biogas output. Table 5 summarizes the results of the kinetic investigation on gas production. It is reported that the Gompertz models matched well with the experimental findings. The late reaction and eventual microorganisms adaptation to the fluctuating atmosphere is expressed in the lag phase (*λ*) [37,46]. The modified Gompertz model have *λ* values of 0.0227 and 0.0287 days, for US 10 min and MW + Fe_3_O_4_, respectively. The calculated values for biogas generation are displayed against the observed values to assess the trustworthiness of the model findings in the tested model. The low RMSE (0.659) and (1.44) data indicate that modified Gompertz can dependably predict high bioactivity. Table 5 lists the statistical indicators (*R*^2^). The higher *R*^2^ (0.996 and 0.993) and lower RMSE values for the modified Gompertz model indicated a more acceptable kinetic model, according to Nguyen et al. [37]. The results showed that the experimental data could be fitted with the modified Gompertz model and could use the model to determine the cumulative biogas production, max biogas potential, maximum biogas production rate and lag time. It is also clear from Figure 13j the biogas data for the predicted and experimental is not fitted well, which is also confirmed by low *R*^2^ (0.858). On the other hand, in Figure 13h, the biogas data for both predicted and experimental is fitted well and in a good arrangement, which is confirmed by high *R*^2^ (0.992).

## 6. Conclusions

In this study, the biomass of green algae *U. intestinalis* was subjected to four pretreatment techniques O_3_, US, MW and Fe_3_O_4_ NPs, either individually or in combination at different doses, to increase its digestibility for processing biogas. The synthesis of green Fe_3_O_4_ was confirmed by various characterization techniques such as TEM, SEM, FTIR and XRD. As a result, in contrast with the untreated biomass studies, the lowest dosages of US, O_3_ (10 min), MW (2 min), and Fe_3_O_4_ (5 mg/L) resulted in the highest biogas yield when sediment is used as a source of anaerobic bacteria. US efficacy in enhancing the solubility of organic matter to increase biogas generation from anaerobic digestion processes or accelerate the digestion of organic matter with reduced time, frequencies and power (10 min—50 Hz and 500 W) has been demonstrated in experimental activities. The findings also suggest that US pretreatment could be useful for lowering digest treatment expenses and increasing biogas generation. According to the energy study, combining MW pretreatment with Fe_3_O_4_ NPs produced more energy while using less input energy than MW pretreatment alone. For each experimental scenario, the kinetic parameters of the reaction were scientifically analyzed using a modified Gompertz function model. The group with the MW pretreatment and Fe_3_O_4_ NPs had a higher potential and maximal biogas generation rate. The shape of the modified Gompertz function model curves indicated that the majority of both the experimental and predicted biogas data was well fitted and confirmed by *R*^2^ and RSME.

## Figures and Tables

**Table 1:** Overview of substrates and pretreatment processes used for the estimation of the biogas yield of *U. intestinalis* in batch experiments.

Experiment	Pretreatment	Incubation Temp. (°C)	I/S Ratio
**Batch 1**	Sediment + algae untreated	37 ± 1	20:1.5
**Batch 2**	Sediment + Algae O_3_ (10 min)	37 ± 1	20:1.5
**Batch 3**	Sediment + Algae O_3_ (15 min)	37 ± 1	20:1.5
**Batch 4**	Sediment + Algae O_3_ (30 min)	37 ± 1	20:1.5
**Batch 5**	Sediment + Algae US (10 min)	37 ± 1	20:1.5
**Batch 6**	Sediment + Algae US (15 min)	37 ± 1	20:1.5
**Batch 7**	Sediment + Algae US (30 min)	37 ± 1	20:1.5
**Batch 8**	Sediment + Algae MW (2 min)	37 ± 1	20:1.5
**Batch 9**	Sediment + Algae MW (4 min)	37 ± 1	20:1.5
**Batch 10**	Sediment + Algae (Fe 5 mg/L)	37 ± 1	20:1.5
**Batch 11**	Sediment + Algae (Fe 10 mg/L)	37 ± 1	20:1.5
**Batch 12**	Sediment + Algae (Fe 20 mg/L)	37 ± 1	20:1.5
**Batch 13**	Sediment + Algae 10 min O_3_ (Fe 5 mg/L)	37 ± 1	20:1.5
**Batch 14**	Sediment + Algae 10 min US (Fe 5 mg/L)	37 ± 1	20:1.5
**Batch 15**	Sediment + Algae 2 min MW (Fe 5 mg/L)	37 ± 1	20:1.5

**Table 2:** EDX spectra of green synthesized Fe_3_O_4_ NPs.

Material	Elements Content	
	O	Fe
**Green Fe_3_O_4_**	18.87 ± 0.6	81.13 ± 1.2

**Table 3:** BET surface area and porosity of green Fe_3_O_4_ NPs.

Sample	BET Surface Area (m^2^/g)	Mean Pore Diameter (nm)	Total Pore Volume (cm^3^/g)
**Green Fe_3_O_4_**	37.85	9.56	0.09

**Table 4:** The proximate values of different substrates.

Proximate Tests	*U. intestinalis*	Sediment
**TS%**	85.11	57.19
**Ash%**	29.45	79.43
**VS%**	70.55	20.57
**C%**	23.05	-
**N%**	2.40	-
**H%**	4.6	-
***C*/*N***	9.60	-

**Table 5:** Data of kinetic analysis using the modified model of Gompertz.

US						
	*R* ^2^	Predicted P (mL/g VS)	Differences (%)	*R*_max_ mL/g VS.day	λ (Day)	RMSE
untreated	0.957	44.14	2.97	9.067	0.54	11.17
10 US	0.987	182.87	2.13	6.18	0.0227	12.11
15 US	0.968	24.83	4.480	3.087	0.298	1.188
30 US	0.984	20.57	2.016	2.71	1.15	0.695
**O_3_**						
untreated	0.957	44.14	2.97	9.067	0.54	11.17
10 O_3_	0.996	187.6	3.42	17.51	0.0890	3.65
15 O_3_	0.985	91.12	7.48	9.47	0.163	3.86
30 O_3_	0.9	86.34	0.22	10.56	0.202	4.84
**MW**						
untreated	0.957	44.14	2.97	9.067	0.54	11.17
2 MW	0.992	83.63	0.55	8.27	0.198	2.66
4 MW	0.926	49.53	8.27	2.38	0.88	3.57
**Fe_3_O_4_**						
untreated	0.957	44.14	2.97	9.067	0.54	11.17
5 Fe_3_O_4_	0.858	35.34	4.55	3.36	0.18	3.71
10 Fe_3_O_4_	0.975	51.63	2.58	3.09	0.35	2.21
20 Fe_3_O_4_	0.969	159.18	1.65	12.26	0.13	10.027
**Combined pretreatments—Fe_3_O_4_**						
US-Fe_3_O_4_	0.951	74.96	1.14	8.85	0.13	5.70
O_3_-Fe_3_O_4_	0.993	70.04	2.71	2.59	0.69	1.44
MW-Fe_3_O_4_	0.979	776.43	7.49	49.89	0.0287	10.14

## Data Availability

Not applicable.

## Sample Availability

Samples of the compounds (O_3_, US and MW treated macroalgae) are available from the authors.

## Abbreviations

AD	Anaerobic digestion
NPs	Nanoparticles
Fe_3_O_4_ NPs	Magnetite nanoparticles
MW	Microwave
O_3_	ozone
US	Ultrasonic
DDW	Double distilled water
FTIR	Fourier transform infrared
XRD	X-ray diffractograms
SEM	Scanning electron microscope
TEM	Transmission electron microscope
EDX	energy dispersive X-ray spectroscopy
BET	Brunauer–Emmett–Teller
TGA	Thermo gravimetric analysis
TS	Total solids
Rm	The maximum biogas production rate
VS	Volatile solids
λ	The lag phase time (days)
